# FLF-RCNN: A Fine-Tuned Lightweight Faster RCNN for Precise and Efficient Industrial Quality Inspection

**DOI:** 10.3390/s26061768

**Published:** 2026-03-11

**Authors:** Ningli An, Zhichao Yang, Liangliang Wan, Jianan Li, Yiming Wang

**Affiliations:** The Faculty of Printing, Packaging Engineering and Digital Media Technology, Xi’an University of Technology, Xi’an 710048, China; 2250820059@stu.xaut.edu.cn (Z.Y.); 2230820068@stu.xaut.edu.cn (L.W.); 2240820063@stu.xaut.edu.cn (J.L.); 2230820035@stu.xaut.edu.cn (Y.W.)

**Keywords:** industrial quality inspection, deep learning, defect detection, fine-tuning, lightweight

## Abstract

Industrial Quality Inspection (IQI) is a pivotal part of intelligent manufacturing, critical to ensuring product quality. Deep learning-based methods have attracted growing attention for their excellent feature extraction ability, outperforming traditional detection approaches. However, existing methods still face issues of insufficient efficiency and poor transferability, and this paper proposes a Fine-tuned Lightweight Faster RCNN (FLF-RCNN) framework designed to address key challenges in IQI, including the trade-off between accuracy and computational efficiency, and the insufficient adaptability of preset anchor box ratios. FLF-RCNN introduces a lightweight backbone network, LSNet, which enhances the receptive field through architectural optimization. Specifically, it uses a collaborative mechanism that combines large kernel convolutions for extracting contextual information and small kernel convolutions for capturing fine-grained details. This mechanism enables the model to efficiently and precisely represent defects. To enhance generalization in data-scarce industrial scenarios, the framework leverages transfer learning with pretrained weights. Furthermore, an Adaptive Anchor Box-Adjustment Module (AAB-AM) based on K-means clustering is introduced to improve detection across varied defect scales. Extensive experiments conducted on the Tianchi dataset show that FLF-RCNN achieves a mAP50 of 43.6%, outperforming detectors using MobileNet and EfficientNet backbones and surpassing the baseline Faster R-CNN by 7.9% in mAP50. Meanwhile, the proposed method reduces computational complexity by approximately 40%, reaching 98.65 GFLOPs, and decreases parameter count by around 30% to 28.2M. These results demonstrate that FLF-RCNN offers a feasibility and practical solution for IQI, achieving a superior accuracy-efficiency balance within the two-stage detection paradigm.

## 1. Introduction

Industrial Quality Inspection (IQI) plays a vital role in ensuring product consistency, enhancing production efficiency, and reducing scrap rates. With the manufacturing industry advancing toward high precision and automation, inspection systems are required to achieve high accuracy, robustness, and computational efficiency [1]. However, existing Deep Learning (DL) methods still struggle to balance accuracy and efficiency in practical industrial deployments [2]. Therefore, improving inference efficiency and deployment efficiency while maintaining detection performance has become a key research challenge.

IQI methods can generally be classified into two categories: traditional image processing techniques (such as edge detection [3], histogram analysis [4], and wavelet transform [5]) and DL-based approaches. Traditional techniques have played a vital role in early IQI tasks, such as the widely adopted Canny edge detection method, known for its high detection accuracy and strong noise resistance [6,7,8]. However, these approaches are heavily reliant on image quality and handcrafted feature design. Consequently, they often lack robustness and generalization capability, making them unsuitable for complex and variable industrial environments. With the rapid advancement of intelligent manufacturing, DL has made remarkable progress in IQI and has been widely applied in various industrial scenarios, such as metal surfaces [9,10], railway tracks [11,12], fabrics [13,14], PCBs [15,16] and engine blades [17,18]. Compared with traditional image processing methods, DL-based end-to-end methods eliminate handcrafted features, enabling better generalization and adaptability across domains.

However, industrial inspection scenarios often involve complex backgrounds, diverse object shapes, and dense small targets, which impose higher demands on the accuracy and robustness of object detection algorithms. Two-stage detectors(such as Faster RCNN [19] and Mask RCNN [20]) incorporate a Region Proposal Network (RPN) and a Region of Interest (RoI) head for refined classification and localization, offering superior accuracy, robustness, and adaptability to challenging backgrounds, often yielding performance that exceeds one-stage methods(such as YOLO [21] and RetinaNet [22]). Nevertheless, their increased structural complexity, larger parameter sizes, and longer inference times hinder efficient deployment in efficiency-constrained industrial systems [23,24]. Although researchers have proposed lightweight backbone networks such as MobileNet [25] and EfficientNet [26] to improve deployment efficiency, these methods often compromise detection accuracy.

Another significant challenge in the industrial domain is the scarcity of high-quality annotated defect datasets. Due to proprietary restrictions and industry competition, publicly available datasets remain extremely limited. Furthermore, the high cost of data annotation and model training has constrained the adoption of DL methods among small- and medium-sized enterprises. These limitations hinder the generalization of models across diverse industrial environments. Consequently, Transfer Learning (TL) based on pretrained models has emerged as a promising solution [27,28,29,30]. By fine-tuning networks that have been pretrained on large-scale general-purpose datasets, it is possible to effectively adapt models to specific industrial tasks, thereby enhancing detection performance while reducing the reliance on extensive labeled data and lowering training costs.

Moreover, the anchor-based mechanisms widely adopted in existing two-stage detection frameworks also current notable limitations [31,32]. These predefined anchors are typically designed based on generic datasets, with fixed scales and aspect ratios that may differ significantly from the geometrical characteristics of industrial defects, such as scratches or cracks. This mismatch can lead to suboptimal candidate region proposals, thereby degrading the overall detection performance, particularly in multi-scale and small object scenarios. Designing more adaptive anchor generation strategies tailored to the geometry of industrial defects is, therefore, a critical direction for improving detection accuracy.

To address the challenges of efficiency optimization and transferability in two-stage detectors, this paper proposes a Fine-tuned Lightweight Faster RCNN (FLF-RCNN). In this context, our efficiency optimization focuses on the two-stage detection paradigm; while not achieving the extreme compactness of single-stage models, FLF-RCNN significantly reduces the computational burden typical of two-stage architectures. We first analyze the variations in defect aspect ratios in real industrial scenarios and design an Adaptive Anchor Box-Adjustment Module (AAB-AM) based on the K-means [33] clustering method. Extensive experiments are conducted on fabric and aluminum datasets released by Tianchi to validate the effectiveness of the proposed method. The main contributions are summarized as follows:

1. We propose the FLF-RCNN computationally optimized framework, which retains the high precision characteristics of two-stage detectors while reducing the overall computation of baseline Faster RCNN from 161.96 Giga Floating-Point Operations Per Second (GFLOPs) to 98.65 GFLOPs, a reduction of approximately 40%. Meanwhile, the number of model parameters is reduced from 41.24 Million (M) to 28.2 M, a decrease of about 30%.
2. To tackle the efficiency bottleneck of two-stage models, we introduce the LSNet backbone, which combines large-kernel convolutions for contextual information extraction and small-kernel convolutions for fine-grained feature extraction, enabling efficient and accurate defect representation within the Faster R-CNN framework.
3. To overcome the difficulty of preset anchor boxes matching the geometric characteristics of industrial defects, an AAB-AM is designed. Compared to the baseline model, this module improves the mAP50 metric by 13.2%. Results on Tianchi dataset demonstrate that FLF-RCNN offers a practical and deployable solution for IQI, balancing detection accuracy with computational efficiency.

The remainder of this paper is organized as follows: Section 2 reviews the related work. Section 3 resents the proposed FLF-RCNN architecture in detail. Section 4 reports and analyzes the experimental results. Finally, Section 5 concludes the paper.

## 2. Related Works

### 2.1. Lightweight Models

To meet the dual requirements of accuracy and efficiency in industrial inspection, researchers have extensively explored lightweight detection networks. These models significantly reduce computational complexity and memory overhead through techniques such as parameter compression [34], structural pruning [35], model quantization [36] and efficient module design [37], thereby achieving strong real-time capabilities. For example, Guan et al. [38] designed a lightweight version of YOLOv7 by integrating techniques such as sparse training, model pruning, and knowledge distillation. This approach reduced the number of parameters by 56.9% while preserving detection accuracy, it achieved an inference speed of 122.7 FPS, which marks an 85% improvement over the original YOLOv7. Song et al. [39] proposed YGNet, which integrates dynamic sparse convolution and a multi-scale feature fusion mechanism. This approach reduces computational cost by approximately 23% in remote sensing object detection tasks, while maintaining a high mAP of 96.2% on the Remote Sensing Object Detection Dataset (RSOD) and achieving an inference speed of 62 Frames Per Second (FPS), demonstrating its potential for deployment in high real-time scenarios. Huang et al. [10] developed a lightweight variant of YOLOv8, named YOLOv8-BVC, in which the original backbone was replaced with VanillaNet. As a result, the model size was reduced by 60 M, the computational cost decreased to 6 GFLOPs, and the inference speed increased to 263 FPS.

Therefore, model lightweighting has significant advantages in reducing computational complexity and improving real-time performance. However, traditional lightweighting methods such as parameter compression, structural pruning, and model quantization often come at the cost of model accuracy: pruning and compression simplify the model by removing redundant structures, which can lead to a decrease in representational capacity; quantization reduces computational overhead by lowering numerical precision, but often introduces noticeable accuracy loss; while knowledge distillation can mitigate performance degradation to some extent, it still struggles to completely avoid accuracy loss. In contrast, designing lightweight networks from scratch allows for the joint optimization of efficiency and accuracy, balancing computational efficiency and feature representation ability during the model construction phase. To this end, this paper introduces LSNet [40], a lightweight backbone network based on efficient convolution design. Through native optimization at the structural level, it significantly reduces computational burden while effectively maintaining high detection accuracy, overcoming the inherent limitations of traditional post-processing lightweighting methods.

### 2.2. Fine-Tuning Methods

To address challenges such as data scarcity and poor task adaptability in IQI, fine-tuned strategies based on pretrained models have attracted significant attention from researchers. These strategies leverage models pretrained on large scale generic datasets and adapt them to specific target tasks through Fine-tuning (FT), effectively enhancing performance in the target domain. For instance, Praveen et al. [27] employed a U-Net architecture combined with a ResNet [41] backbone pretrained on ImageNet for steel surface defect segmentation. This TL approach significantly improved classification accuracy by 5% and segmentation precision by 26%, with especially notable gains in low-data scenarios. Cheng et al. [28] proposed a two-stage training process along with a “metric reweighting module” to tackle class imbalance, achieving a substantial performance boost of up to 11.98% in detecting rare aluminum defect categories. Seungmi et al. [29] designed an anomaly detection system that estimates the density of normal data by FT a feature extractor, enabling effective domain distribution learning and improved anomaly detection performance without additional computational overhead. Woo-Kyun et al. [30] developed a fine-tuned MobileNet1-SC model that achieved superior detection results on the StitchingNet dataset. These examples demonstrate that TL-based FT method not only alleviates the data insufficiency commonly faced in industrial inspection tasks but also shortens training cycles and enhances model robustness and generalization capabilities. As a result, FT has become a critical technical route in intelligent industrial inspection. Inspired by these methods, this paper uses fine-tuned strategies into a lightweight detection framework and adapts it to the characteristics of industrial tasks for further evaluation.

### 2.3. Anchor-Based Detection Method

Anchor-based detection methods have been widely adopted in object detection frameworks such as Faster RCNN [19] and RetinaNet [22], owing to their stable training and reliable performance, where the Faster RCNN uses anchors for prediction proposals, while the RetinaNet is used for dense prediction. The two-stage method maintains high performance in complex scenarios due to its anchor prediction proposal-based feature. However, in industrial scenarios where defects often exhibit irregular shapes, small sizes, or extreme aspect ratios, the fixed anchor configurations, often optimized for general-purpose datasets like COCO, may poorly align with the geometric characteristics of industrial defects may not provide adequate coverage. This mismatch can result in low recall rates and poor localization, especially for small or elongated targets. To address this, some studies have introduced K-means clustering to optimize anchor ratios, while others propose data-driven or dynamic anchor mechanisms such as Meta Anchor and Guided Anchoring [31,32].

Nevertheless, these improvements may increase training complexity or remain insufficient under highly variable industrial conditions. In this work, we retain the anchor-based paradigm and enhance anchor adaptability through a K-means-based adjustment strategy tailored to defect geometries, thereby enhancing multi-scale defect detection without introducing additional structural complexity.

## 3. Methodology

### 3.1. FLF-Framework

**Figure 1:**
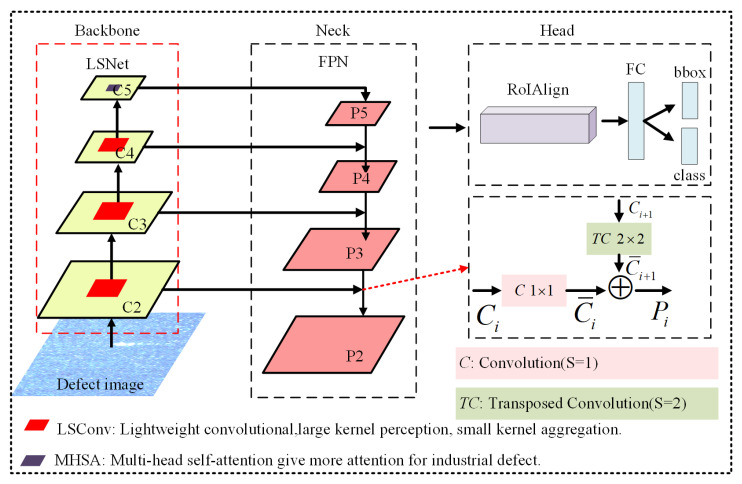
FLF-RCNN architecture with an LSNet backbone using LSConv and multi-head attention for multi-scale feature extraction(C2–C5), an FPN neck with initial transposed convolution for fine-grained features, and a head for defect classification and localization.

This paper proposes FLF-RCNN, a lightweight object detection framework based on Faster RCNN that is specifically designed for industrial quality inspection (IQI) tasks. As illustrated in Figure 1, the architecture consists of backbone, neck and head. To achieve model lightweight, the traditional feature extraction backbone is replaced by a lightweight network, which is LSNet. LSNet adopts LSConv as its fundamental building block, which optimizes the receptive field of convolutional kernels by leveraging a collaborative mechanism of large kernel convolutions for contextual information and small kernel convolutions for fine-grained details. This design enables efficient and accurate defect feature modeling. In contrast to traditional lightweight methods that focus on pruning, parameter compression, or sparsification, LSNet achieves reduced computational complexity and model size by introducing a streamlined and optimized network architecture. It achieves superior inference efficiency without compromising detection performance, making it particularly suitable for IQI scenarios where deployment efficiency is critical. Moreover, this work incorporates a fine-tuned strategy by adapting the pretrained backbone from large-scale generic datasets to the target industrial task. This TL approach enhances the model’s generalization ability and practical applicability in diverse industrial inspection environments.

### 3.2. LSConv

**Figure 2:**
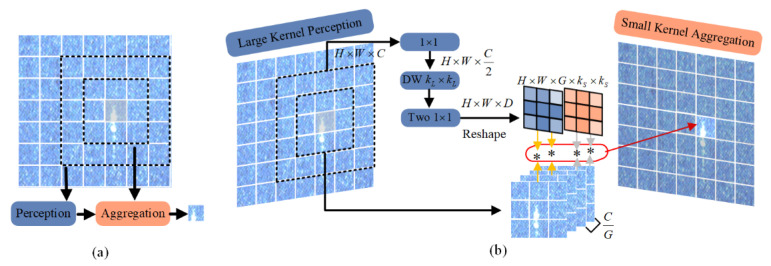
Large Kernel Perception (LKP) and Small Kernel Aggregation (SKA) in LSConv: (**a**) Collaborative mechanism combining large-kernel context perception and small-kernel detail aggregation; (**b**) Computational details showing depth-wise large-kernel convolution for contextual weights and grouped dynamic convolution for feature aggregation. Here, * denotes the point-wise convolution.

The LSConv is designed to overcome the limitations of traditional convolution operations such as fixed kernel weights and the computational redundancy associated with self-attention mechanisms. By employing large convolutional kernels to capture broader contextual information and small kernels for localized feature aggregation, LSConv facilitates efficient information fusion. This approach allows computational resources to be focused on defect regions, thereby achieving model lightweight without sacrificing performance. Where represents the output of this region. By adjusting the positions of perception and aggregation, the model enables contextual representation across varying receptive fields, denoted as ${ℜ}_{P}\left(x_{i}\right)$ and ${ℜ}_{A}\left(x_{i}\right)$. This mechanism allows for capturing both global contextual information and fine-grained local details at different scales, thereby enhancing the representation of defect features. For regions requiring only limited contextual aggregation, an adaptive weighted feature summation strategy is employed to reduce computational overhead. As illustrated in Figure 2a, the core idea involves using large kernels for broad contextual perception and small kernels for localized aggregation. This “see large, focus small” design effectively balances accuracy and efficiency. In the following paragraph, we provide a detailed formulation of computation process.

**Large Kernel Perception (LKP)**: The LKP adopts the design of large kernel bottleneck block. As shown on the left side of Figure 2b, the token is first projected to a lower channel dimension through point-wise convolution (PW) to achieve lightweight. By adopting large kernel Depth-Wise convolution (DW) with a kernel size of $k_{L}\times k_{L}$, the large field of view spatial contextual information of ${ℜ}_{k}\left(x_{i}\right)$ is effectively captured. Where ${ℜ}_{k}\left(x_{i}\right)$ represents the convolution calculation area of size $k_{L}\times k_{L}$ centered on $x_{i}$. This method can improve the expression ability of context at the lowest cost and generate contextual adaptive weights $W\in R^{H\times W\times D}$ for the feature aggregation process. This process can be expressed as:
  (1) $$\omega_{i}=P_{ls}\left(x_{i},{ℜ}_{k_{L}}\left(x_{i}\right)\right)=\mathrm{PW}(DW_{k_{L}\times k_{L}}(\mathrm{PW}({ℜ}_{k_{L}}\left(x_{i}\right))))$$
  where $\omega_{i}\in R^{D}$ is the generation weight of $x_{i}$.

**Small Kernel Aggregation (SKA)**: The SKA adopts the design of grouped dynamic convolution, as shown on the right side of Figure 2b. By dividing the channels into *G* groups, each group containing C/G channels and the channels in the same group share the aggregation weights to reduce the memory overhead and computational cost of the lightweight model. Then the weight $\omega_{i}\in R^{D}$ of $x_{i}$ obtained by perception also needs to be processed to obtain $\omega_{i}^{*}\in R^{G\times k_{S}\times k_{S}}$, where $k_{S}\times k_{S}$ is the size of the aggregated convolution kernel. The context ${ℜ}_{k_{S}}\left(x_{i}\right)$ is aggregated through $\omega_{i}^{*}$, where ${ℜ}_{k_{S}}\left(x_{i}\right)$ represents the neighborhood of size $k_{S}\times k_{S}$ centered on $x_{i}$. Thus, the aggregated feature is obtained $y_{i}^{*}$. This process can be expressed as:
  (2) $$y_{i}^{*}=A_{ls}\left(\omega_{ig}^{*},{ℜ}_{k_{S}}\left({x_{i}}_{c}\right)\right)=\omega_{ig}^{*}*{ℜ}_{k_{S}}\left({x_{i}}_{c}\right)$$

### 3.3. LSNet

**Figure 3:**
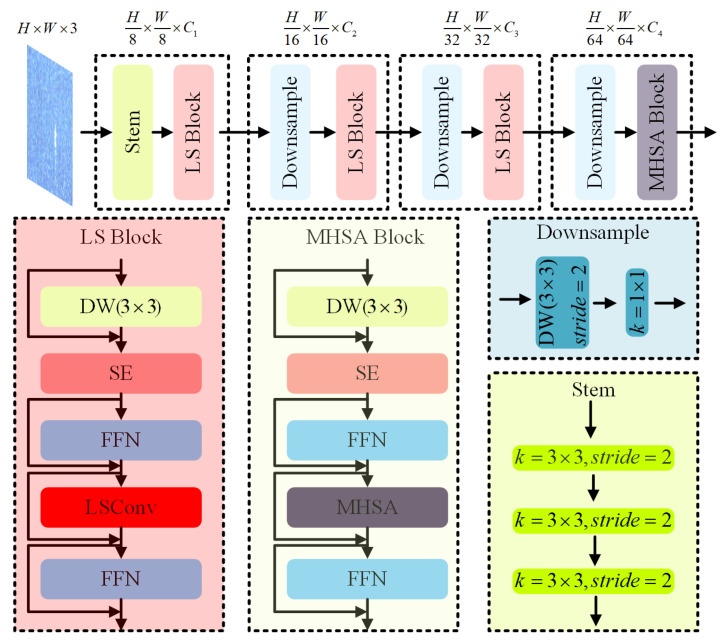
Architecture of the adopted LSNet backbone. The network comprises four stages: Stage 1 uses the Stem module for initial downsampling and semantic extraction; Stages 2–3 employ LS Blocks with LSConv core units for multi-scale feature extraction via a collaborative large/small kernel mechanism; Stage 4 introduces an MHSA module for global context aggregation. Within the LS Block, spatial-contextual weights from Large Kernel Perception (LKP) and channel-wise weights from the SE module operate at distinct levels (spatial perception vs. channel recalibration) and are applied sequentially. Residual connections and FFN are integrated to enhance convergence and nonlinear expression.

LSNet consists of four components: Stem, LS Block, Down-sample, and MHSA Block. Its structure is shown in Figure 3. Among them, LS Block uses LSConv as the core building unit, combines the feedforward network (FFN) to enhance the nonlinear expression ability, and introduces an SE module to adaptively adjust channel-wise weight. It is worth noting that the spatial-contextual weights generated by LKP within LSConv and the channel-wise attention weights from the SE module operate at distinct levels—spatial perception versus channel recalibration—and are applied sequentially to complementarily enhance feature representation. The network accelerates convergence and optimization through residual connections. LSNet is divided into four stages: In the first stage, the Stem module performs preliminary downsampling to reduce feature map size and extract basic semantic information. Subsequently, LS Blocks are introduced for feature extraction, combining depthwise separable convolution and pointwise convolution to achieve spatial downsampling and channel compression. The second and third stages repeat this combination of downsampling and LS Blocks to further deepen the network. To enhance the modeling of long-range dependencies in low-resolution feature maps, a Multi-Head Self-Attention (MHSA) module is introduced in the fourth stage, enabling more effective global context aggregation.

### 3.4. Fine-Tuning

In practical DL applications, training a deep neural network from scratch typically requires a large amount of labeled data and considerable computational resources, which poses significant challenges in industrial visual inspection tasks. To address this, FT has emerged as an efficient TL strategy and is widely adopted for model adaptation. The core idea of FT is to initialize the model with weights pretrained on large-scale general-purpose datasets, and then update all or part of the parameters to align with the specific characteristics of the target domain. This process enables the model to quickly adapt to new dataset distributions and task requirements, thereby improving convergence speed and generalization ability.

Specifically, let the parameters of the pretrained model be denoted as θ, which has already acquired strong feature representation capabilities on a large-scale source dataset $D_{s}$. When adapting the model to a new target dataset $D_{t}$, we initialize the model with θ and define the FT objective as minimizing the loss function over $D_{t}$, formulated as:

(3) $$\theta^{*}=\arg\min_{\theta }L\left(\theta ,D_{t}\right)$$

where $L(\theta ,D_{t})$ denotes the task specific loss function on the target domain. The FT process is equivalent to starting from a well-trained initialization point θ in the parameter space and making slight adjustments to the model parameters using data from the target task. This strategy supports knowledge transfer and task-specific adaptation, effectively addressing the limitations of data scarcity and insufficient generalization faced by models trained from scratch.

In this study, we incorporate the LSNet, pretrained on a general object detection dataset, as the backbone feature extractor of the Faster RCNN framework. A staged unfreezing strategy is adopted for FT, where LSNet is divided into four stages. The parameters are progressively unfrozen, beginning with the feature extraction layers and ultimately FT the entire network, thereby enabling a systematic analysis of the impact of various FT strategies on model performance.

## 4. Experiments

### 4.1. Datasets Description

**Figure 4:**
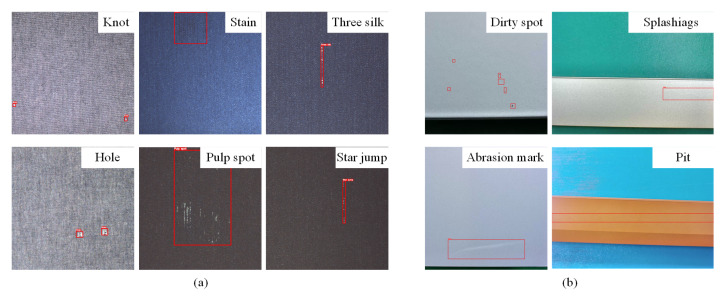
Defect examples in Fabric and Aluminum datasets. (**a**) Fabric defect examples; (**b**) Aluminum defect examples.

To verify the effectiveness of the proposed FLF-RCNN model, we conducted a systematic evaluation on the Tianchi fabric dataset (Fabric) [42] and the aluminum surface defect dataset (Aluminum) [43]. Figure 4 shows representative defect samples from the two datasets (cropped to highlight the defect area). It can be observed that there are obvious differences between the two datasets in terms of target size and anchor box ratio. Specifically, the defects in the Fabric dataset are usually small in size, slender in shape, and have blurred edges, which makes them difficult to detect; while the defects in the Aluminum dataset are characterized by uneven size distribution and complex and diverse shapes. Thus, we explain the composition of the two datasets respectively, and perform parameter adjustment and model evaluation based on their characteristics in subsequent experiments.

**Figure 5:**
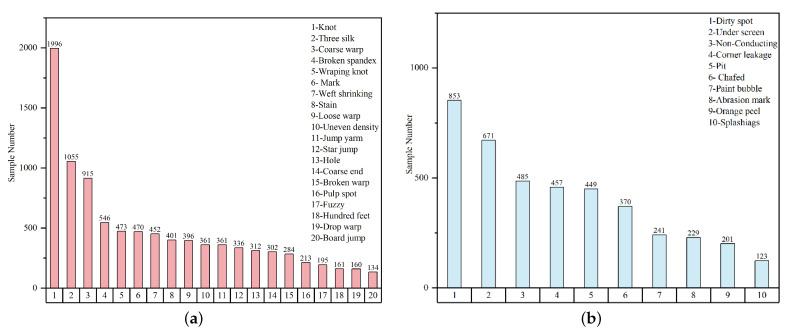
Statistical results of Tianchi (**a**) fabric dataset and (**b**) aluminum dataset.
**Aluminum**: This dataset contains 3005 images with a resolution of 2560×1920. There are 10 types of defects and 4079 labeled instances. The detailed statistics are shown in Figure 5b.

**Fabric**: This dataset has a total of 5913 images, each with a resolution of 2446×1000, and a total of 20 types of defects. We counted each defect on each image, a total of 9523 defects, and the detailed statistical data set for each type of defect is shown in Figure 5a.

**Figure 6:**
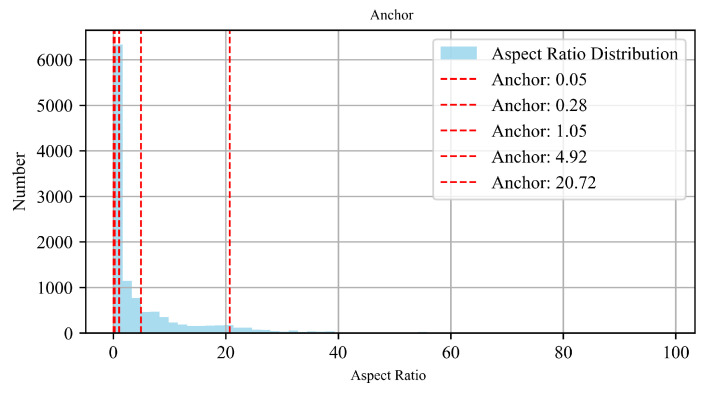
Aspect ratio distribution. The red dashed line represents the anchor box value automatically generated by K-means method.

To improve the detection model’s adaptability to varying geometric characteristics of targets, we conducted a statistical analysis of the aspect ratios of all defect targets across two datasets, as shown in Figure 6, the results reveal significant variations in aspect ratios with a long-tail distribution. To address the insufficient adaptability of predefined anchor boxes, this study employs the K-means clustering algorithm to automatically generate anchor candidate ratios that better fit the real aspect ratios of targets. The specific procedure is as follows, let the set of all target aspect ratios be $X=\{x_{1},x_{2},\ldots ,x_{N}\}$, to reduce clustering bias caused by scale differences, the natural logarithm of the aspect ratios is first computed, resulting in:

(4) $$r_{i}=\log x_{i},i=1,2\ldots ,N$$

where $r_{i}$ represents the result of applying the logarithm to $x_{i}$, forming a new set $\overset{⌢}{X}$ = {r1,r2,…rN}. Then, we perform K-means clustering on $\overset{⌢}{X}$, partitioning the data into *K* clusters, which are defined as:

(5) $$\min_{C_{1},C_{2},\ldots C_{K}}\sum_{k=1}^{K}{\sum_{r_{i}\in C_{k}}∥r_{i}-\mu_{k}∥}^{2}$$

Among them, $C_{K}$ represents the point set of the number of *k*, and $\mu_{k}$ represents the center of the *k*th cluster. After clustering, an exponential inverse transformation is applied to the cluster centers to obtain the set of anchor box ratios used in practice:

(6) $$Tr=\exp\;(\mu_{k})$$

Finally, we get a set of anchor box ratios $A=\{Tr_{1},Tr_{2},\ldots Tr_{N}\}$. The red dashed lines in represent the adaptive anchor box ratios generated by the proposed method. This approach dynamically adjusts anchor parameters according to the target shapes in different datasets, thereby enhancing the detector’s adaptability to multi-scale defects.

### 4.2. Implementations Details

The network is implemented in Python 3.8 under the Pytorch framework. The experiment is built on a Linux system and uses an Nvidia GeForce RTX 2080Ti GPU. The hyperparameter settings used in the experiment are shown in Table 1, where including learning rate, batch size, number of training rounds, anchor box ratio generates by AAB-AM, the number of clusters *K*, large kernel $k_{L}$ and small kernel $k_{s}$, all of which are obtained through manual tuning to ensure the stability and convergence of model performance. To ensure the fairness of the comparative experiments, all the comparative methods use the official implementation provided by the open source target detection tool library MMDetection [44] in the comparison of the whole methods, in order to fairly evaluate the contribution of the lightweight backbone LSNet introduced in this paper to the model performance, all the two-stage methods used for comparison are embedded with AAB-AM. The specific contribution of each component will be analyzed in detail in the ablation experiment.

### 4.3. Evaluation Metrics

To compare the model effects more fairly, two common indicators, precision and recall, were used in the experiments, which are defined as:

(7) $$P=\frac{TP}{TP+FP}$$

(8) $$R=\frac{TP}{TP+FN}$$

Among them, *P* represents precision, *R* represents recall, TP, FP, and FN represent the number of true positives, false positives, and false negatives, respectively. IoU is the core indicator for judging whether the predicted bounding box matches the ground truth bounding box, which directly determines the positive and negative sample division of the detection result. It is defined as:

(9) $$IoU=\frac{AreaofOverlap(Pb\cap Gt)}{AreaofUnion(Pb\cup Gt)}$$

where Pb represents the predicted box and Gt represents the ground truth bounding box. Whether the predicted bounding box is a positive sample needs to be determined based on the predetermined threshold of IoU. If the IoU threshold is 0.5, all bounding boxes with IoU greater than 0.5 are positive samples, and the rest are negative samples. In this case, the calculated AP value is defined as AP50, and AP75 and other indicators have similar definitions. We will not make too much quantitative description. Here, we give the definitions of mAP and mAR:

(10) $$mAP=\frac{1}{n}\sum_{i=1}^{n}AP_{i}$$

(11) $$mAR=\frac{1}{n}\sum_{i=1}^{n}AR_{i}$$

where *i* represents the average accuracy of a specific class and *n* represents the number of classes. In addition, since the defect scales of real industrial data vary, to compare the performance of the model at different defect scales, we briefly introduce mAP_s, mAP_m and mAP_l. Given the definition of defect area:

(12) $$defectarea=\left|\left(x_{1}-x_{2}\right)\times \left(y_{1}-y_{2}\right)\right|$$

where $x_{1},x_{2}$ represent the two horizontal coordinates of the ground truth, and $y_{1},y_{2}$ represent the two vertical coordinates of the ground truth.

If $defectarea<32^{2}$, then:

(13) mAP_s=mAP

If $32^{2}<defectarea<96^{2}$, then:

(14) mAP_m=mAP

If $defectarea>96^{2}$, then:

(15) mAP_l=mAP

### 4.4. Comparative Experiment

To comprehensively evaluate the lightweight performance and IQI capability of the proposed FLF-RCNN model, we conducted comparative experiments on the Tianchi dataset with some representative object detection models, encompassing both one-stage and two-stage approaches [45,46,47]. The experiments first assessed the parameter count (Param) and computational complexity (FLOPs) of each model on the fabric dataset, with detailed results shown in Table 2 and corresponding performance metrics presented in Table 3. Subsequently, detection accuracy and other performance indicators were evaluated on the aluminum dataset, as summarized in Table 4.

As shown in Table 2, FLF-RCNN demonstrates significant advantages in model complexity compared to most two-stage detectors and some one-stage detectors such as YOLOv3, YOLOV5l and YOLOV7. While maintaining architectural integrity, FLF-RCNN achieves a notable reduction in both parameter count and computational cost, highlighting its lightweight characteristics. Although there remains a parameter gap compared to lightweight models such as MobileNet and EfficientNet. Table 3 reveals that FLF-RCNN significantly outperforms these models in detection accuracy, reflecting a superior balance between accuracy and efficiency. Notably, FLF-RCNN enhances detection performance while reducing model complexity, primarily through the optimized its perception and aggregation mechanisms. Specifically, compared to the baseline Faster RCNN, FLF-RCNN achieves a 7.9% improvement in mAP and a 13% gain in mAP50. It exhibits particularly strong performance in detecting large targets, with a 10% increase in mAP_l. In terms of model complexity, its computational cost is reduced from 161.96 GFLOPs to 98.65 GFLOPs (a reduction of approximately 40%), and parameter count decreases from 41.24M to 28.2M (around 30% reduction).

On the aluminum dataset, FLF-RCNN achieves the best overall performance. As shown in Table 4, it outperforms all compared models on most key evaluation metrics. Since most defect targets in this dataset are relatively large, the mAP_s is marked as “Nan,” which aligns with the data distribution characteristics.

**Figure 7:**
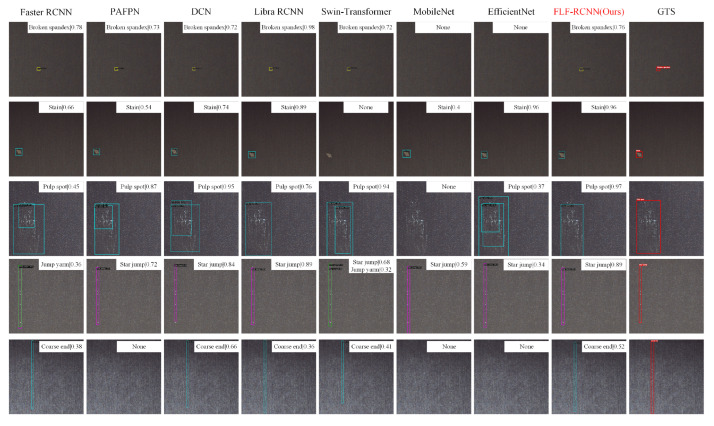
Qualitative comparison of detection results on the Fabric dataset for five representative defect categories: Broken Spandex, Stain, Pulp Spot, Star Jump, and Coarse End. Each row shows detection outputs from different methods (Faster RCNN, PAFPN, DCN, Libra RCNN, Swin-Transformer, MobileNet, EfficientNet and FLF-RCNN). FLF-RCNN demonstrates superior localization accuracy and reduced false positives, particularly for small-scale, slender, or edge-blurred defects where other methods exhibit missed detections or imprecise bounding boxes.

**Figure 8:**
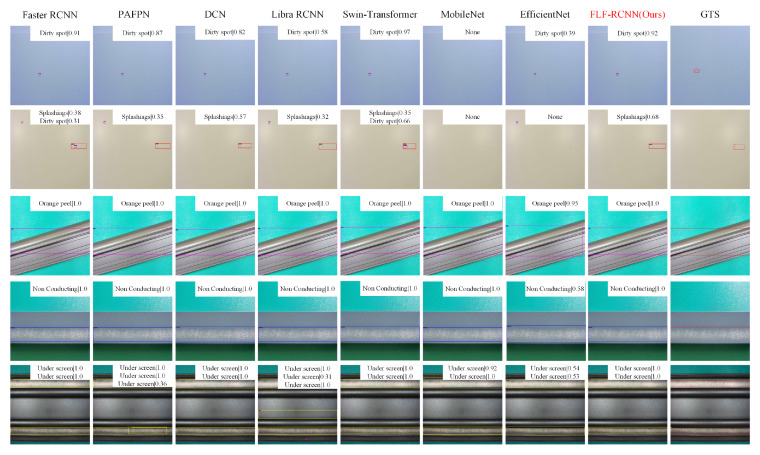
Qualitative comparison of detection results on the Aluminum dataset. The dataset features defects with irregular shapes and varying scales. FLF-RCNN achieves more accurate and consistent detection frames with fewer redundant proposals compared to two-stage methods and one-stage detectors, validating its robustness to diverse industrial defect morphologies.

Furthermore, we conducted a visual analysis of the detection results for selected models. Representative detection outputs are presented in Figure 7 and Figure 8. Figure 7 illustrates the detection performance of various models on five representative defect categories: Broken Spandex, Stain, Pulp Spot, Star jump, and Coarse End. The results reveal considerable performance variation across different models and defect types. Specifically, for Broken Spandex defects, both MobileNet and EfficientNet exhibit systematic missed detections. In the case of Stain defects, Swin-Transformer fails to detect any instances, whereas other models maintain stable performance, highlighting the Transformer-based architecture’s sensitivity to low-contrast anomalies. For Pulp spot, multiple methods have multiple overlapping bounding boxes, suggesting difficulties in precise localization under complex texture backgrounds. Regarding Coarse End detection, all models except PAFPN, MobileNet, and EfficientNet successfully identify the defects.

Overall, FLF-RCNN demonstrates superior perform on small scale (such as Broken Spandex and Stain), slender in shape (such as Jump Yarn and Coarse End), and blurred edges (such as Pulp Spot). This performance gain is primarily attributed to the LSConv in the feature extraction stage. The large kernel convolution component effectively expands the receptive fields and enhances the model’s ability to capture global contextual semantics, which is particularly beneficial for detecting slender structural defects. Meanwhile, the small kernel convolution component, equipped with an adaptive weighted feature fusion mechanism, strengthens the modeling of local texture and fine-grained details. The collaborative mechanism between these two components enable LSNet to achieve accurate localization and identification, even in challenging industrial scenarios involving complex textures, small targets, or severely deformed defects.

On the aluminum dataset, FLF-RCNN also performed well, with accurate detection frames, less redundancy, and fine adaptability to defects of different sizes, further verifying the feasibility and practical value of this method in complex industrial scenarios.

### 4.5. Ablation Experiment

To verify the practical effects of the key components in the proposed model, we designed and conducted a systematic ablation experiment on the Tianchi fabric dataset. The specific results are shown in Table 5 and Table 6.

As shown in Table 5, first, we evaluated the impact of the AAB-AM on detection performance. Experimental results indicate that the application of the K-means-based AAB-AM leads to a notable 3.0 percentage point improvement in the mAP_l metric (from 9.1% to 12.1%), with additional performance gains observed across other evaluation metrics. This demonstrates that reconstructing anchor box ratios according to the scale diversity of defects effectively enhances the model’s ability to match multi-scale targets, thereby improving overall detection accuracy. Subsequently, we assessed the effectiveness of the incorporated computationally optimized backbone network, LSNet [38]. By replacing the conventional ResNet backbone with LSNet, we observed that the model achieved the highest scores across all metrics except mAP_m (medium object). These improvements are attributed to LSNet’s optimized receptive field and feature aggregation strategies, which enable more comprehensive extraction of multi-scale defect features and more accurate representation and localization of defects.

To further evaluate the effectiveness of the fine-tuned strategy, we conducted a stage-wise unfreezing experiment, as shown in Table 6. Based on the LSNet backbone, two training strategies were evaluated: random initialization and transfer learning with fine-tuning. Specifically, the stage-wise unfreezing strategy progressively unlocks backbone layers from deeper stages (Stage 4) to shallower stages (Stage 1), while maintaining a uniform learning rate (0.0001) across all layers to ensure stability. The results reveal that models trained from random initialization exhibit significantly lower detection accuracy. In contrast, under the TL setting, progressively unfreezing the backbone network led to a steady improvement in model accuracy as more layers were unlocked. The model achieved optimal performance under the full fine-tuning configuration (no frozen layers). These results demonstrate that the fine-tuned strategy based on a pretrained model not only effectively retains general visual representations but also allows the model to gradually adapt to the specific patterns of industrial inspection tasks. This facilitates faster convergence and improves training stability, which is critical for data-scarce industrial scenarios.

**Figure 9:**
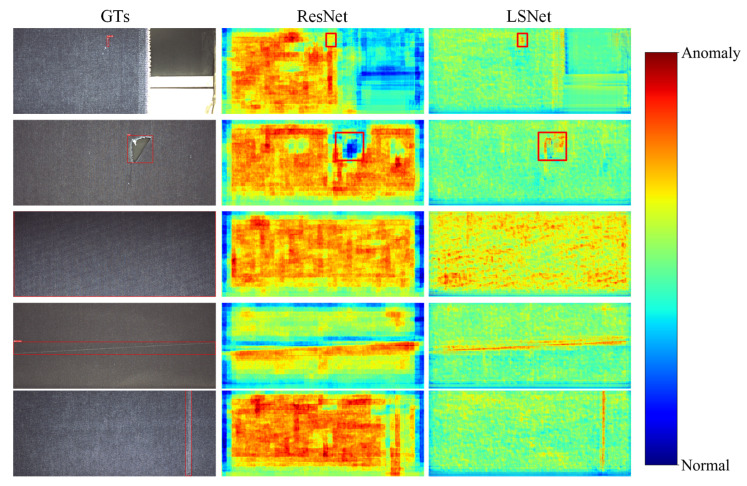
Feature response visualization on representative defect samples: (**a**) ResNet backbone produces diffuse activations with significant background noise; (**b**) LSNet exhibits focused activation on defect regions with suppressed background interference, demonstrating superior spatial selectivity for industrial defect detection.

Furthermore, to verify the feature representation capability of LSNet, we compared its feature response maps with those of ResNet at the first layer output of the FPN, as illustrated in Figure 9. Visualization of five representative defect samples-characterized by small size, slender shape and blurred edges, reveals that ResNet produces significant background noise and lacks precise focus on defect regions. In contrast, LSNet exhibits superior spatial selectivity, with strong activation in defect regions and significantly suppressed background interference, enabling accurate localization of the defects. Whether dealing with tiny, slender, or edge-blurred defects, LSNet consistently provides effective feature representations. The visual discrepancy between the two methods clearly demonstrates the advantage of LSNet in both feature perception and expression. This benefit stems from its cooperative large kernel and small kernel receptive field structure and efficient feature aggregation strategy.

In summary, FLF-RCNN achieves a well-balanced trade-off between accuracy and efficiency by integrating AAB-AM and a fine-tuned lightweight backbone. Without relying on destructive compression techniques such as pruning or quantization, the model achieves approximately a 40% reduction in computational cost and a 30% reduction in parameter count, while improving detection accuracy (mAP) by up to 7.9%. These results highlight the high deployment potential of the proposed model, especially in real-time industrial inspection scenarios with limited computing resources.

## 5. Conclusions

In this paper, a computationally efficient detection method, termed FLF-RCNN, is proposed for IQI. Built upon the Faster R-CNN architecture, FLF-RCNN is designed to maintain high detection accuracy while significantly reducing computational complexity and parameter size relative to standard two-stage detectors.

Specifically, a fine-tuned optimized backbone network, LSNet, is incorporated as the feature extractor. By leveraging large-kernel convolution for contextual information extraction and small-kernel convolution for efficient feature aggregation, LSNet achieves approximately 30% parameter reduction and approximately 40% FLOPs reduction without sacrificing representation capability. Additionally, an AAB-AM module based on K-means clustering is introduced, which improves the detection performance for large-scale defects (mAP_l) by 3%. Overall, the proposed FLF-RCNN outperforms the baseline Faster R-CNN by 7.9% in mAP. Compared with typical lightweight models, although FLF-RCNN still has a gap in parameter size compared to architectures such as MobileNet and EfficientNet, its superior mAP performance demonstrates its practical value in high-precision IQI scenarios where detection accuracy is prioritized over extreme model compactness.

Future work will focus on further validating the generalization capability of the proposed framework across more diverse industrial defect domains. In particular, we plan to extend our method to other surface defect benchmarks such as the SSGD dataset [52], and adapt the framework to better accommodate material-specific characteristics. We also intend to explore model compression and optimization strategies to further enhance deployment efficiency in resource-constrained industrial environments.

## Figures and Tables

**Table 1:** Hyperparameter settings.

Parameters	Value
Batch size	2
Epoch	12
Lr	0.0001
Weight decay	0.05
*K*	5
Anchor box ratio	[0.05,0.28,1.05,4.92,20.72]
$k_{L}$	7
$k_{s}$	3

**Table 2:** Comparison of model parameters and computational complexity.

Stage	Model	Param (M)	Flops (G)
One	YOLOV3 [45]	61.95	9.766
	YOLOV3 + MobileNet [25]	4.23	6.046
	YOLOV5s [45]	13.7	16.4
	YOLOV5l [45]	89.0	114.2
	YOLOV7 [45]	74.8	103.4
	RetinaNet + EfficientNet [26]	18.73	109.3
Two	Faster RCNN [19]	41.24	160.96
	PAFPN [48]	44.78	179.7
	DCN [49]	97.22	168.03
	Libra RCNN [47]	41.5	161.76
	JDCBL [13]	43.69	160.21
	FCOS-AMFF [50]	83.9	414.1
	Faster-AMFF [50]	76.11	400.8
	RetinaNet-AMFF [50]	88.36	420.1
	Swin-Transformer [51]	44.85	165.6
	FLF-RCNN (Ours)	28.2	98.65

**Table 3:** Experimental results on fabric dataset. The best metrics are highlighted in bold.

Stage	Model	${\mathrm{mAP}}_{0}$	mAP50	${\mathrm{mAP}}_{5}\%$	${\mathrm{mAP}}_{m}$	${\mathrm{mAP}}_{1\%}$	${\mathrm{mAP}}_{10\%}$
One	SSD [46]	5.6	15.9	7.0	7.1	1.9	–
	YOLOV3 [46]	9.3	27.5	7.1	9.2	7.3	–
	MobileNet [25]	3.7	11.1	3.2	1.2	3.2	12.4
	RetinaNet [13]	7.2	18.2	–	–	–	–
	RetinaNet-AMFF [50]	17.7	41.1	11.9	15.9	21.4	–
	EfficientNet [26]	11.6	29.4	10.6	10.2	10.0	32.8
Two	Faster RCNN [19]	12.0	29.6	12.6	13.5	9.1	24.4
	PAFPN [48]	13.5	32.1	11.5	13.2	12.7	25.9
	DCN [49]	13.3	33.6	14.8	15.0	13.1	27.3
	Libra RCNN [47]	11.6	28.3	13.9	13.2	12.1	21.9
	JDCBL [13]	15.6	37.2	16.0	15.6	12.7	28.4
	FCOS-AMFF [50]	17.8	38.0	14.2	**18.5**	23.3	–
	Faster-AMFF [50]	17.5	39.1	15.7	18.2	**23.8**	–
	Swin-Transformer [51]	15.4	34.6	15.1	14.1	15.1	30.1
	FLF-RCNN (Ours)	**19.9**	**43.6**	**17.1**	14.2	19.1	**37.9**

**Table 4:** Experimental results on aluminium dataset. The best metrics are highlighted in bold.

Stage	Model	${\mathrm{mAP}}_{0}$	${\mathrm{mAP}}_{50}$	${\mathrm{mAP}}_{75}$	${\mathrm{mAP}}_{s}$	${\mathrm{mAP}}_{m}$	${\mathrm{mAP}}_{l}$	${\mathrm{mAP}}_{m}$	${\mathrm{mAP}}_{l}$
One	MobileNet [25]	31.3	53.8	Nan	0.2	44.3	46.7	–	–
	RetinaNet [13]	34.1	58.3	Nan	3.4	36.1	–	–	–
	RetinaNet-AMFF [50]	35.5	61.2	Nan	2.9	27.4	–	–	–
	EfficientNet [26]	28.7	55.4	Nan	1.8	37.1	46.8	–	–
Two	Faster RCNN [19]	34.7	48.9	Nan	4.7	35.6	48.7	–	–
	PAFPN [48]	35.7	48.7	Nan	1.3	35.1	48.7	–	–
	DCN [49]	37.8	53.4	Nan	1.4	36.5	49.6	–	–
	Libra RCNN [47]	35.2	51.3	Nan	1.9	35.2	49.5	–	–
	GPTNet [17]	32.6	47.0	Nan	–	–	–	38.9	–
	SPGNN [18]	34.5	50.4	Nan	4.3	50.4	42.6	–	–
	FCOS-AMFF [50]	38.5	61.5	Nan	5.0	39.6	–	–	–
	Faster-AMFF [50]	36.5	50.5	Nan	4.8	37.3	–	–	–
	Swin-Transformer [51]	36.2	59.8	Nan	7.4	38.4	46.3	–	–
	FLF-RCNN (Ours)	**55.3**	**74.8**	Nan	**9.4**	**58.6**	**62.2**	–	–

**Table 5:** Ablation Analysis on Fabric. × denotes ablation-free, ✓ denotes ablation. The best metrics are highlighted in bold.

AAB-AM	LSNet	mAP	mAP50	mAP75	mAP_s	mAP_m	mAP_l	mAR
×	×	12.0	29.6	9.8	12.6	13.5	9.1	24.4
✓	×	16.6	36.4	12.7	16.6	**17.5**	12.1	34.1
✓	✓	**19.9**	**43.6**	**15.3**	**17.1**	14.2	**19.1**	**37.9**

**Table 6:** The impact of frozen layers on model performance. The best metrics are highlighted in bold.

Backbone	Stage	mAP	mAP50	mAP75	mAR
LSNet	Random Initialization	2.5	6.5	1.2	7.1
	(1,2,3,4)	13.7	31.2	9.3	29.1
	(1,2,3)	13.9	33.9	9.4	29.8
	(1,2)	17.9	40.2	12.6	33.9
	(1)	18.9	41.1	15.1	36.3
	None	**19.9**	**43.6**	**15.3**	**37.9**

## Data Availability

The data presented in this study are available on request from the corresponding author. The data are not publicly available due to privacy.
